# Nutrient content prediction and geographical origin identification of red raspberry fruits by combining hyperspectral imaging with chemometrics

**DOI:** 10.3389/fnut.2022.980095

**Published:** 2022-10-17

**Authors:** Youyou Wang, Yue Zhang, Yuwei Yuan, Yuyang Zhao, Jing Nie, Tiegui Nan, Luqi Huang, Jian Yang

**Affiliations:** ^1^State Key Laboratory Breeding Base of Dao-di Herbs, National Resource Center for Chinese Materia Medica, China Academy of Chinese Medical Sciences, Beijing, China; ^2^School of Traditional Chinese Medicine, Yunnan University of Chinese Medicine, Kunming, China; ^3^Institute of Agro-product Safety and Nutrition, Zhejiang Academy of Agricultural Sciences; Key Laboratory of Information Traceability for Agricultural Products, Ministry of Agriculture and Rural Affairs of China, Hangzhou, China

**Keywords:** red raspberry, hyperspectral imaging, chemometrics, nutrients content, geographic origin, prediction

## Abstract

The geographical origin and the important nutrient contents greatly affect the quality of red raspberry (RRB, *Rubus idaeus* L.), a popular fruit with various health benefits. In this study, a chemometrics-assisted hyperspectral imaging (HSI) method was developed for predicting the nutrient contents, including pectin polysaccharides (PPS), reducing sugars (RS), total flavonoids (TF) and total phenolics (TP), and identifying the geographical origin of RRB fruits. The results showed that these nutrient contents in RRB fruits had significant differences between regions (*P* < 0.05) and could be well predicted based on the HSI full or effective wavelengths selected through competitive adaptive reweighted sampling (CARS) and variable iterative space shrinkage approach (VISSA). The best prediction results of PPS, RS, TF, and TP contents were achieved with the highest residual predictive deviation (RPD) values of 3.66, 3.95, 2.85, and 4.85, respectively. The RRB fruits from multi-regions in China were effectively distinguished by using the first derivative-partial least squares discriminant analysis (DER-PLSDA) model, with an accuracy of above 97%. Meanwhile, the fruits from three protected geographical indication (PGI) regions were successfully classified by using the orthogonal partial least squares discrimination analysis (OPLSDA) model, with an accuracy of above 98%. The study results indicate that HSI assisted with chemometrics is a promising method for predicting the important nutrient contents and identifying the geographical origin of red raspberry fruits.

## Introduction

Red raspberry (RRB, *Rubus idaeus* L.), a woody plant of the genus *Rubus* in the family Rosaceae, has recently become a popular fruit in the market. Because of their sweet taste and unique flavor, RRB fruits are often processed into canned food, jam, jelly, juice, fruit wine, etc. (1). The pleasant flavor and taste in fruits are mainly affected by the contents of reducing sugars (RS), including fructose, sucrose, glucose, etc. (2, 3). RRB fruits are rich in healthy nutrients, including pectin polysaccharides (PPS) (4), total flavonoids (TF) (5), and total phenolics (TP) (2). Also, RRB fruits have many health-promoting benefits, such as antioxidant, anti-inflammatory (6), and anti-cancer effects (2, 7).

Accurate and efficient evaluation of the nutrient contents (RS, PPS, TF, and TP) is of great significance for symbolizing the edible and nutraceutical quality of fruits. However, all these indicators are usually evaluated with some destructive, time-consuming, and costly chemical methods, including high-performance liquid chromatography (HPLC) and mass spectrometry (MS) (5, 8).

In recent years, RRB fruits are popular in some East Asian countries, such as China, Japan, and South Korea (7, 9). In China, Dexing County of Jiangxi Province (JXDX), Chun’an County (ZJCA), and Lishui City (ZJLS) of Zhejiang Province have long planting histories. Attributed to abundant rainfall and sufficient illumination, these areas become famous for the high quality of RRB fruits. Dexing raspberry, Chun’an raspberry, and Lishui raspberry have won the protected geographical indications (PGI), and the protected areas reached 10,000 hm^2^ in 2019, yielding about 7,500 tons of dry products for output (10, 11). In legislation, the PGI can identify products from protected regions and ensure a high-quality reputation in the market.

However, many illegal cases such as geographical origin and brand counterfeiting for higher profits occur frequently in the RRB market. Unfortunately, the conventional and destructive methods for RRB fruit origin identification, including DNA bar code and element and chemical fingerprints, suffer from a long pretreatment cycle and high cost (8, 12).

Considering the above problems, it is urgent to develop non-destructive, inexpensive, and time-saving methods for predicting the nutrient contents and geographical origin of RRB fruits, thus ensuring fruit quality. As a non-destructive and rapid detection method, hyperspectral imaging (HSI) technology can provide the spectral reflectance of any pixel at hundreds of wavebands. It can assess many samples at one time without any pre-treatment and has been widely used in fruit quality evaluation (13–15).

By combining HSI with chemometrics, researchers have efficiently predicted the RS content in fruits of blueberries (15) and pomelo (16), as well as the PPS content in mulberry fruit and orange peels (17, 18). Meanwhile, the total flavonoids (TF) and total phenolics (TP) contents in black goji berries and grape fruits were successfully estimated, indicating the great potential in nutrient content prediction based on full/selected HSI wavelengths (15, 19). Moreover, in geographical origin traceability using HSI full/selected wavelengths combined with chemometric models, narrow-leaved oleaster (*Elaeagnus angustifolia*) fruits (13), Chinese wolfberries (20), and banana (*Musa* spp.) fruits (21) were identified with high prediction accuracies. However, to the best of our knowledge, there have been no reports of its application to RRB fruits.

This study aimed to investigate the feasibility of detecting the nutrient content and identifying the geographical origin of RRB fruits using HSI combined with chemometrics. The specific objectives were: (a) To determine the nutrient content differences in RS, PPS, TF, and TP of RRB fruits from multiple production regions; (b) To evaluate the performance of predicting nutrient contents based on full/selected HSI wavelengths, and (c) To reveal the authenticity of origins, especially PGI status, of RRB fruits by using HSI technology combined with chemometrics.

## Materials and methods

### Sample collection and preparation

RRB fruits were collected from nine provinces in August 2020, covering all ten main production regions in China (Supplementary Table 1). In this study, 30 healthy mature fruits (about 50 g) with uniform size and color were treated as one subsample (30 fruits) for HSI data collection. In each production region, 10 sampling plots were set and 5 replicates of subsamples were collected from each plot. So that each production region has 50 (10 plots × 5 replicates) subsamples to obtain locally representative parallel HSI data. All the harvested subsamples were immediately stored in ice chilled chest coolers at 4°C and transported as fresh food to the laboratory by aircraft at 4°C. The samples were analyzed for completion of HSI spectra collection within 48 h to minimize the impact from the different periods for transporting (22). After HSI analysis, fruits in each of the 500 subsamples (50 subsamples × 10 regions) from 10 main production regions were immediately homogenized into fruit pulp by a homogenizer (PB206A, Midea, Guangzhou, China). The obtained fruit pulp from each subsample was further treated for nutrient contents measurement through conventional chemical methods with a spectrometer, so as to build a reference database for evaluation of the HSI prediction effect (23).

### Hyperspectral imaging system and spectral information extraction

A visible and short-wave/long-wave near-infrared hyperspectral imaging spectrometer (VIS-NIR-HSI, HySpex VNIR-1800/HySpex SWIR 384, Norsk Elektro Optikk, Oslo, Norway) was employed to obtain spectral information for prediction analysis. The HSI is comprised of two tungsten halogen lamps (150 W/12 V, H-LAM Norsk Elektro Optikk, Oslo, Norway), and VNIR (350–990 nm, H-V16, Norsk Elektro Optikk, Oslo, Norway) and SWIR (900–2,550 nm, H-S16, Norsk Elektro Optikk, Oslo, Norway) lenses with a spectral resolution of about 5 nm. The distance between the lenses and samples was 25 cm, and the moving speed of the platform was 2.5 mm/s. To avoid obvious noise fluctuations at the start and the end of the wavelengths, only the collected effective spectral information, including 396 bands from 410 to 950 nm and from 950 to 2,500 nm, was merged manually with the two lenses. Furthermore, to eliminate the adverse influence of external factors such as uneven light distribution and camera dark current, the HSI data was corrected before further analysis with the following correction formula:


$$R=\left(R_{r\,a\,w}-R_{d}\right)/\left(R_{w}-R_{d}\right)$$

where *R* is the corrected spectral data, *R*_*raw*_ is the original spectral data, *R*_*w*_ is the white reference data obtained from the white board with a reflectivity of 99%, and *R*_*d*_ is the dark reference data obtained by turning off the light and blocking the camera lenses. The spectral information of each fruit was treated as one region of interest (ROI) and extracted using the ENVI 5.3 software (Harris Geospatial Solutions Inc., CO, USA). Then, all the pixel reflectance data were calculated to obtain the average of one subsample.

### Reference measurement of red raspberry nutrients content

#### Measurement of pectin polysaccharides content

According to the requirements of the extraction kit (YX-W-ZDT, Hepeng Biological, Shanghai, China) and the phenol-sulfuric acid method, the PPS solution extracted from the fruit pulp was measured using the Multiskan SkyHigh-1510 microplate spectrophotometer (Thermo Fisher, MA, US) at 490 nm (4). Meanwhile, standard samples of glucose (99% in purity, YX-W-ZDT, Hepeng Biological, Shanghai, China) at concentrations of 1, 0.5, 0.25, 0.125, and 0.0625 mg/ml were prepared to construct a standard curve with the square of curve correlation coefficient (R^2^) value equal to 0.9992 (y = 0.0212x-0.0103). Then, the PPS content was calculated according to the standard curve.

#### Measurement of reducing sugars content

RS can reduce 3,5-dinitrosalicylic acid (DNS) reagent in alkaline solutions, and the red-brown precipitate product can be assessed at 540 nm with a microplate spectrophotometer. According to the instruction of the detection kit (BC2710, Solarbio, Beijing, China), the RS extracted from the RRB fruit pulp was reacted with DNS, and glucose solutions (99% in purity, YX-W-ZDT, Hepeng Biological, Shanghai, China) at concentrations of 1, 0.8, 0.5, 0.2, and 0.1 mg/ml were used as standard samples to construct a standard curve with R^2^ value equal to 0.9997 (y = 0.0211x-0.0088). Then, the content of RS was calculated based on the standard curve.

#### Evaluation of total flavonoids content

In alkaline nitrite solution, flavonoids and aluminum ions will form a red complex with an obvious absorption at 470 nm (4). According to the instruction of the extraction kit (BC1330, Solarbio, Beijing, China), reference solutions of rutin (98% in purity, BC1330, Solarbio, Beijing, China) at concentrations of 1.5, 1.25, 0.625, 0.3125, 0.15625, 0.078, 0.039, and 0.02 mg/ml were prepared to construct a standard curve with R^2^ value equal to 0.9993 (y = 0.021x-0.0099). Then, the content of TF extracted from the RRB fruit pulp was calculated according to the standard curve.

#### Assessment of total phenolics content

Under alkaline conditions, phenols can reduce tungstomolybdic acid to produce blue compounds with a characteristic absorption peak at 760 nm (6). According to the instruction of the extraction kit (BC1340, Solarbio, Beijing, China), standard samples of gallic acid (98% in purity, BC1340, Solarbio, Beijing, China) at concentrations of 0.15625, 0.078125, 0.039, 0.02, 0.01, 0.005, and 0.0025 mg/ml were prepared to construct a standard curve with R^2^ value equal to 0.9996 (y = 0.0183x-0.0024). Then, the content of TP extracted from fruit pulp was calculated based on the standard curve.

### Statistical and chemometrics analysis

#### Statistical analysis

The data of nutrient contents obtained with chemical methods were applied to significant difference analysis between regions (*P* < 0.05). One-way analysis of variance (ANOVA) using Duncan’s multiple comparison method was implemented on the SPSS software (22.0 version, IBM Inc., Chicago, IL, USA). All significant difference analysis results were expressed as mean ± standard deviation of three replicates.

#### Model prediction of nutrients content based on hyperspectral imaging wavelengths

Four pretreatment methods, including the first derivative (DER), the second derivative (SEC), multiplicative signal correction (MSC), and Savitzky-Golay filtering (SG) with a window size of 9, were adopted to eliminate random interferences and improve the HSI spectral features and performance. Three prediction models used in this study are briefly described below, including back-propagation neural network (BPNN), partial least square regression (PLSR), and support vector machine (SVM).

The BPNN, as a widely used method in regression, always has three or more neurons, including an input layer, hidden intermediate layers, and an output layer (Supplementary Figure 1). During the analysis, the activation function value of the neuron is delivered through these layers in the mentioned order. Based on the difference between actual and prediction values, the weight values are corrected layer by layer from the output layer to the input layer. In our BPNN model, the node number of the hidden layers was set at 10, and the momentum factor, initial weight, and the learning step were adjusted to 0.3, 0.95, and 0.1, respectively. The maximum training iteration was adjusted to 100, and the minimum error was adjusted to 0.001 (23).

The PLSR model is a classical linear regression algorithm. It can consider both matrices x (spectral data) and y (chemical index) and find the maximal correlation between the new variables of X and Y (24, 25). In our analysis, the leave-one-out cross-validation method was adopted to obtain the optimal number of important latent variables ranging from 6 to 10 in different nutrient prediction groups using PLSR.

The SVM model is suitable for analyzing both linear and non-linear data, and it has the advantages of less training time, lower computation complexity, and better generalization ability. In this research, the SVM model was constructed based on the radial basis function, and the optimal combination of two important parameters, i.e., the penalty factor (C, ranging from 2^–8^ to 2^8^) and the kernel parameter (γ, ranging from 100 to 2,500), were determined through a grid-search method. Also, the influence of sampling randomness on model performance was greatly avoided by leave-one-out cross-validation so as to improve the efficiency and accuracy of parameter optimization (26).

The prediction effect of pretreatment methods combined with regression models was evaluated based on residual predictive deviation (RPD) and curve correlation coefficient R^2^ values. Usually, the R^2^ value from 0.61 to 0.80 and the RPD from 2.0 to 2.5 indicate that the model can be used for prediction; the R^2^ value from 0.81 to 0.90 and the RPD value from 2.5 to 3.0 indicate high model performance; the R^2^ value higher than 0.90 and the RPD value higher than 3.0 indicate excellent model performance (4).

#### Effective wavelengths selection for nutrients content prediction

Two wavelength selection methods were adopted in this study, including competitive adaptive reweighted sampling (CARS) and variable iterative space shrinkage approach (VISSA). The CARS method can evaluate the importance of each variable. A two-step method was adopted to select the key variables, including (a) forced variable selection based on an exponential decline function, and (b) competitive variable selection based on adaptive reweighted sampling. Meanwhile, three parameters, including the maximal principle to extract, the group number for cross-validation, and the pretreatment method, were set to 10, 10, and “autoscaling,” respectively.

The VISSA method has two important rules during optimization: (a) the variable space shrinks in each step, and (b) the core of the VISSA model is that a new variable space is superior to the previous one. The performance of variable space in each optimization step could be evaluated, and the weighted binary matrix sampling method was used in this model to generate sub-models to span the variable subspace (27). In VISSA model selection, four parameters, including the maximum number of latent variables, the group number for cross-validation, the number of binary matrix sampling, and the pretreatment method, were set to 15, 10, 500, and “autoscaling,” respectively.

#### Models for geographical origin traceability of red raspberry based on full wavelengths

In this study, two discrimination models (PLSDA and SVM) in combination with four pre-treatment methods (DER, MSC, SEC, and SG) were adopted for geographical origin tracing. The PLSDA model projected the prediction variables and observation variables into a new space to find a linear regression for multi-origin classification. In the PLSDA model, the latent variables ranging from 6 to 10 were determined by leave-one-out cross-validation. The SVM model, which aims to obtain the best hyperplane by selecting the hyperplane passing through the maximum possible gap between points of different categories, was used with a non-linear radial basis function to reduce the training complexity. In SVM model, the penalty factor (C) and kernel parameter (γ) are two important parameters for improving the accuracy of the radial basis function, and they were selected through a grid search algorithm (the same above in section “Model prediction of nutrients content based on hyperspectral imaging wavelengths”). The performance of the models in geographical origin identification was evaluated in terms of sensitivity, specificity, and accuracy. These three indices were calculated as follows:


Accuracy(%)=100(TP+TN)*/(TP+TN+FP+FN)

Sensitivity(%)=100T*P/(TP+FN)

Specificity(%)=100T*N/(TN+FP)

where *TP, TN, FN*, and *FP* represent the numbers of true positives, true negatives, false negatives, and false positives, respectively (28).

The samples from three PGI regions (ZJLS, ZJCA, and JXDX) were pairwise compared with those from common regions by using the OPLS-DA model based on the full HSI wavelengths, and 200 permutation tests were conducted to avoid any over-fitting problem. Discrimination accuracy, R^2^X, R^2^Y, and Q^2^ were recorded to represent the classification efficiency, the explanatory power for the variation in X variables and Y variables, and the predictive capability of the model, respectively.

**FIGURE 1:**
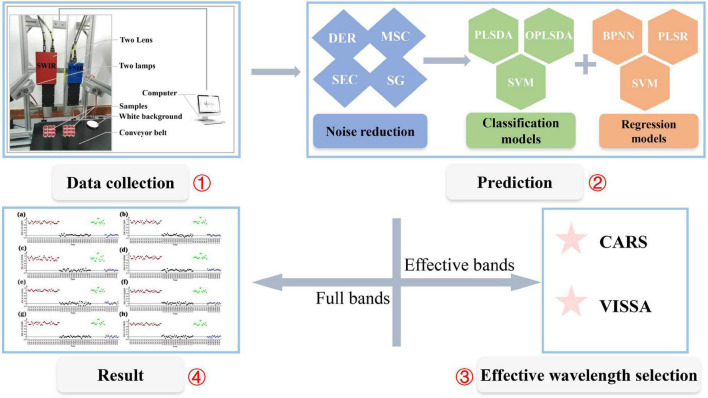
The specific process of the analysis. The workflow includes four parts, i.e., data collection, indicators prediction, effective wavelengths selection, and results analysis. Four pretreatment methods were considered, including the first derivative (DER), multiplicative signal correction (MSC), the second derivative (SEC), and Savitzky-Golay filtering (SG). Three regression models, including back-propagation neural network (BPNN), partial least square regression (PLSR), and support vector machines (SVM), were used for contents prediction. Three classification models, including orthogonal partial least squares discrimination analysis (OPLSDA), partial least squares discriminant analysis (PLSDA), and support vector machines (SVM), were used for origins prediction. Two wavelength selection methods were considered, including competitive adaptive reweighted sampling (CARS) and variable iterative space shrinkage approach (VISSA).

In both nutrient content and origin prediction, all the RRB samples were grouped into a prediction set and a training set at the ratio of 3:7 using the SPXY algorithm (i.e., sample set partitioning based on joint x-y distances) (29). Also, all the above models were implemented by using MATLAB software (R2020a, The MathWorks, Inc., MA, USA). The specific analysis workflow of the study is shown in Figure 1.

## Results

### Analysis of nutrients content for red raspberry fruits from different geographical origins

The measured PPS, RS, TF, and TP contents of RRB fruits are listed in Supplementary Table 1. The PPS content ranged from 63 mg/g (GZQDN) to 227 mg/g (AHXC). The RS content ranged from 119 mg/g in the JXDX region to 339 mg/g in the ZJLS region. As for the TF content, the maximum and minimum values were 34.0 mg/g (AHXC) and 3.0 mg/g (GXYL), respectively. Besides, the maximum (14.2 mg/g) and the minimum values (10.0 mg/g) of the TP content were found in the JXDX region and the ZJLS region, respectively. The results showed that the mean contents of the four nutritional indicators had a significant difference (*P* < 0.05) among the most different production regions (Supplementary Table 1). Overall, PPS and RS contents account for 10–20 and 20–30 in RRB fruits, respectively, and both represent the total sugar content in the fruits. The TF and TP contents account for 1–3 and 1–2% of the total weight, respectively.

### Prediction of nutrients content based on full wavelengths

In this part, three regression models (BPNN, PLSR, and SVM) were combined with pretreatment measures including DER, MSC, SEC, and SG, respectively, to predict the nutrient contents. The results showed that some combinations of pretreatment and regression models performed well on the prediction sets (Supplementary Tables 2–5). In PPS prediction, the PLSR model obtained desirable results, with RPD values of 2.5–3.0. Meanwhile, the DER-SVM group showed a good ability for PPS prediction, with an RPD value of 2.55 (Supplementary Table 2). As for RS prediction, the DER-PLSR, MSC-PLSR, SEC-PLSR, and SG-PLSR groups exhibited an excellent ability, with R^2^ values above 0.90 and RPD values above 3.0 (Supplementary Table 3). In TF content prediction, only the DER-PLSR and MSC-PLSR groups performed well, with RPD values of 2.51 and 2.55, respectively (Supplementary Table 4). As for TP prediction, the groups of DER-PLSR, MSC-PLSR, SEC-PLSR, and SG-PLSR showed an excellent ability, with R^2^ values above 0.90 and RPD values higher than 3.0. The DER-SVM group also had an excellent ability for TP content prediction, with an R^2^ value above 0.90 and an RPD value of 3.11 (Supplementary Table 5).

### Prediction of red raspberry nutrients content based on selected hyperspectral imaging wavelengths

In this part, according to the prediction results based on full HSI wavelengths, some models with good or excellent prediction effects were further adopted to select important variables. Specifically, the models were ORI-PLSR, DER-PLSR, MSC-PLSR, SEC-PLSR, SG-PLSR, and DER-SVM (RPD > 2.5) for PPS prediction (Supplementary Table 2), DER-PLSR, MSC-PLSR, SEC-PLSR, and SG-PLSR (RPD > 3) for RS prediction (Supplementary Table 3), DER-PLSR and MSC-PLSR (RPD > 2.5) for TF prediction (Supplementary Table 4), and DER-PLSR, MSC-PLSR, SEC-PLSR, SG-PLSR, and DER-SVM (RPD > 3) for TP prediction (Supplementary Table 5). Meanwhile, two strategies of CARS and VISSA with different characteristics were used for wavelength selection.

The nutrient content prediction using selected wavelengths via the CARS method (Table 1) obtained higher RPD values (in bold, 13 out of 17 groups) than those from the full wavelength groups. Specifically, the RPD values were improved from the level (from 2.5 to 3.0) indicating a good ability to the best level (>3.0) indicating an excellent ability in PPS content prediction (Table 1). As for using the VISSA method in wavelength selection, the good results showed that the RPD values increased in about 10 out of 17 groups (RPD values in bold, Table 2).

**TABLE 1:** Nutrient content prediction based on the effective wavelengths selected by the CARS method.

Nutrients	Wavelengths number	Models	Training set		Prediction set		
			R^2^	RMSET	R^2^	RMSEP	RPD
PPS	86	ORI-PLSR	0.926	12.21	0.899	12.26	**3.66**
	80	DER-PLSR	0.925	12.28	0.912	13.30	**3.39**
	113	MSC-PLSR	0.939	11.08	0.883	15.27	2.87
	85	SEC-PLSR	0.902	14.10	0.879	13.14	**3.36**
	83	SG-PLSR	0.907	13.67	0.880	12.99	**3.47**
	80	DER-SVM	0.959	9.54	0.881	15.10	**2.82**
RS	92	DER-PLSR	0.939	8.27	0.904	12.17	2.90
	83	MSC-PLSR	0.954	7.21	0.949	8.78	**3.95**
	80	SEC-PLSR	0.939	8.32	0.926	10.20	**3.47**
	94	SG-PLSR	0.904	10.40	0.905	12.17	2.91
TF	104	DER-PLSR	0.913	2.49	0.851	3.11	2.47
	93	MSC-PLSR	0.923	2.35	0.866	2.94	2.67
TP	83	DER-PLSR	0.963	0.195	0.958	0.238	**4.69**
	118	MSC-PLSR	0.973	0.167	0.961	0.229	**4.85**
	102	SEC-PLSR	0.961	0.198	0.956	0.245	**4.56**
	100	SG-PLSR	0.964	0.162	0.959	0.239	**4.72**
	83	DER-SVM	0.996	0.064	0.954	0.251	**4.42**

CARS, competitive adaptive reweighted sampling method for wavelength selection. PPS, RS, TF, and TP indicate pectin polysaccharides, reducing sugars, total flavonoids, and total phenolics, respectively. R^2^, square of curve correlation coefficient; RMSET, root mean square error on the training group; RMSEP, root mean square error on the prediction group; RPD, residual predictive deviation. RPD values in bold represent the improvement of model performance compared with that from the full wavelengths group. Also, the RPD values with underline indicate the best model group for this nutrient content regression. ORI, original spectrum; Four pretreatment methods include DER, first derivative; SEC, second derivative; SG, Savitzky-Golay filtering; MSC, multiplicative signal correction; Three regression models include BPNN, back-propagation neural network, PLSR, partial least square regression; SVM, support vector machines. The same abbreviations are used below.

**TABLE 2:** Nutrient content prediction based on the effective wavelengths selected by the VISSA method.

Nutrients	Wavelengths number	Models	Training set		Prediction set		
			R^2^	RMSET	R^2^	RMSEP	RPD
PPS	110	ORI-PLSR	0.871	16.11	0.868	16.45	**2.74**
	121	DER-PLSR	0.922	12.58	0.874	15.87	**2.79**
	146	MSC-PLSR	0.905	13.88	0.849	17.29	2.50
	102	SEC-PLSR	0.903	13.99	0.881	15.86	**2.89**
	118	SG-PLSR	0.903	13.98	0.866	16.51	**2.71**
	121	DER-SVM	0.973	7.92	0.885	14.79	**2.83**
RS	110	DER-PLSR	0.950	7.50	0.933	9.80	**3.75**
	90	MSC-PLSR	0.907	8.68	0.933	12.14	2.68
	112	SEC-PLSR	0.930	8.90	0.906	11.75	3.03
	88	SG-PLSR	0.927	9.10	0.909	11.61	2.92
TF	120	DER-PLSR	0.947	1.94	0.882	2.75	**2.85**
	97	MSC-PLSR	0.894	2.75	0.801	3.60	2.16
TP	127	DER-PLSR	0.952	0.222	0.917	0.350	3.16
	114	MSC-PLSR	0.927	0.271	0.909	0.368	2.82
	125	SEC-PLSR	0.961	0.200	0.935	0.295	**3.77**
	110	SG-PLSR	0.957	0.208	0.942	0.287	**3.87**
	127	DER-SVM	0.999	0.028	0.918	0.341	**3.19**

VISSA, variable iterative space shrinkage approach for wavelength selection.

Generally, compared with full wavelength groups, CARS-ORI-PLSR and CARS-MSC-PLSR were the most successful methods for PPS and RS content prediction, with the highest RPD values of 3.66 and 3.95 respectively, indicating an excellent ability in prediction (RPD values with underline, Table 1). The CARS-MSC-PLSR was the most efficient method for TP content prediction, with the highest RPD value of 4.85, indicating an excellent ability in prediction (RPD values with underline, Table 1). As for TF content prediction, the most suitable model VISSA-DER-PLSR obtained the highest RPD value of 2.85, indicating a good ability in prediction (RPD values with underline, Table 2).

### Identification of the geographical origin of red raspberry fruits using hyperspectral imaging full wavelengths

**FIGURE 2:**
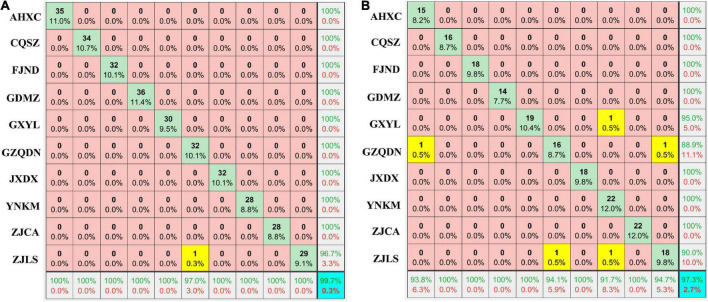
Geographical origins discrimination for RRB fruits using the DER-PLSDA model based on full wavelengths. Training set **(A)**, Prediction set **(B)**. The number in blue color represents the total discrimination accuracy on the training and prediction sets, and the yellow color represents the groups with discrimination errors. The results of sensitivity and specificity are shown in the bottom row and the right column, respectively.

The PLSDA and SVM models combined with pre-treatment methods were used in the geographical origin discrimination of RRB fruits (Table 3). Overall, the PLSDA model combined with pre-treatment methods had a better classification effect than the SVM group, and the total discrimination accuracy was higher than 89% on both the training and prediction sets (Table 3). In the PLSDA group with pre-treatment, all discrimination accuracy was improved when compared with that of the ORI group without pretreatment (Table 3). In this study, the DER-PLSDA model exhibited the highest total discrimination accuracy of 99.7 and 97.3% in the training and prediction groups, respectively (Table 3 and Figure 2). As shown on the training set (Figure 2A) and prediction set (Figure 2B), the sensitivity, specificity, and discrimination accuracy of the two PGI regions of ZJCA and JXDX were all 100%, while those of the ZJLS region were 100, 96.7, and 96.7 on the training set (Figure 2A) and 94.7, 90.0, and 90.0% on the prediction set, respectively (Figure 2B).

**TABLE 3:** Multi-origin discrimination of fruits using PLSDA and SVM models.

Pretreatments	PLSDA		SVM	
	Training (%)	Prediction (%)	Training (%)	Prediction (%)
ORI	81.1	78.1	64.6	59.3
DER	99.7	97.3	85.4	66.7
MSC	95.3	92.9	62.0	56.0
SEC	96.9	89.1	82.6	70.7
SG	93.4	90.7	50.3	44.0

PLSDA, partial least squares discriminant analysis; SVM, Support vector machines.

Then, the RRB fruits from PGI status regions (ZJLS, ZJCA, and JXDX) were classified using the OPLSDA model based on the full HSI wavelengths, and the prediction accuracy is shown in Table 4 and Supplementary Figures 2–4. The parameter of Q^2^ indicates the predictive ability, where 0.9 > Q^2^ > 0.5 represents a good ability for prediction, and Q^2^ ≥ 0.9 indicate an excellent predictive ability (30). In the groups of ZJLS vs. the others, the discrimination accuracy was 100%, and the Q^2^ values were all higher than 0.9, except for the groups of ZJLS vs. ZJCA with Q^2^ equal to 0.887 (Table 4 and Supplementary Figure 2). In the discrimination of ZJCA samples, the accuracy was 100%, and the Q^2^ values were all higher than 0.9 (Table 4 and Supplementary Figure 3). However, several misjudgment cases occurred in discrimination of JXDX samples (Table 4 and Supplementary Figure 4). In the groups of JXDX vs. AHXC, JXDX vs. CQSZ, JXDX vs. GXYL, JXDX vs. GZQDN, and JXDX vs. YNKM, discrimination errors were observed, the accuracy ranged from 98 to 99%, and the Q^2^ values ranged from 0.80 to 0.90. In the groups of JXDX vs. FJND and JXDX vs. GDMZ, the discrimination accuracy was both 100%, and the Q^2^ values were 0.960 and 0.841, respectively (Table 4 and Supplementary Figure 4).

**TABLE 4:** Pairwise discrimination of fruits from PGI regions using the OPLSDA model.

Groups	R^2^X	R^2^Y	Q^2^	Accuracy (%)
ZJLS vs. AHXC	0.825	0.975	0.973	100
ZJLS vs. CQSZ	0.933	0.981	0.981	100
ZJLS vs. FJND	0.904	0.955	0.949	100
ZJLS vs. GDMZ	0.989	0.975	0.973	100
ZJLS vs. GXYL	0.937	0.972	0.968	100
ZJLS vs. GZQDN	0.989	0.977	0.975	100
ZJLS vs. JXDX	0.959	0.972	0.970	100
ZJLS vs. YNKM	0.924	0.97	0.969	100
ZJLS vs. ZJCA	0.975	0.917	0.887	100
ZJCA vs. AHXC	0.920	0.977	0.973	100
ZJCA vs. CQSZ	0.870	0.973	0.968	100
ZJCA vs. FJND	0.837	0.947	0.938	100
ZJCA vs. GDMZ	0.912	0.973	0.968	100
ZJCA vs. GXYL	0.938	0.976	0.974	100
ZJCA vs. GZQDN	0.929	0.976	0.974	100
ZJCA vs. JXDX	0.924	0.976	0.973	100
ZJCA vs. YNKM	0.939	0.977	0.973	100
JXDX vs. AHXC	0.992	0.933	0.898	99
JXDX vs. CQSZ	0.944	0.863	0.858	99
JXDX vs. FJND	0.960	0.965	0.960	100
JXDX vs. GDMZ	0.979	0.881	0.841	100
JXDX vs. GXYL	0.770	0.842	0.829	99
JXDX vs. GZQDN	0.982	0.894	0.846	98
JXDX vs. YNKM	0.984	0.848	0.819	99

PGI, protected geographical indication; OPLSDA, orthogonal partial least squares discrimination analysis. R^2^X and R^2^Y are recorded to exhibit the explanatory power for the variation in X variables and Y variables, respectively. Q^2^ represents the predictive capability of the model. 0.9 > Q^2^ > 0.5 indicates a good ability for prediction, and Q^2^ ≥ 0.9 indicates an excellent predictive ability. vs., versus.

## Discussion

Fruit nutrients such as PPS, RS, TF, and TP are important indicators in quality evaluation. Similar studies have been reported on the nutrient content prediction of fruits using a fast and non-destructive method, providing evidence of good prediction effects when HSI was combined with chemometric methods. For example, NIR-HSI combined with the PLSR model was successfully applied to the prediction of RS content in pomelo fruits (16) as well as the PPS content in mulberry fruits (18) and orange peels (17), and low RMSE values and high R^2^ values were obtained. Meanwhile, in a former report, the TP and TF contents from black goji berries were effectively determined using HSI full wavelengths combined with PLSR and SVM models (15).

Selecting some key wavelengths instead of using the full wavelengths can reduce model complexity and improve prediction accuracy and robustness. Similar to our results, many relevant reports demonstrated that the nutrient content prediction results in fruits based on the selected HSI wavelengths are similar to or better than those from the full-band group. For example, in the prediction of the TF and TP contents in black goji berries based on the effective wavelengths selected via CARS and successive projections algorithm (SPA) methods, the R^2^ and RPD values were the same as those from the full-band group (15). In the prediction of total anthocyanin content in mulberry fruits (31) and sugar content in pomelo fruits (32), better prediction results were obtained in effective wavelength groups.

**FIGURE 3:**
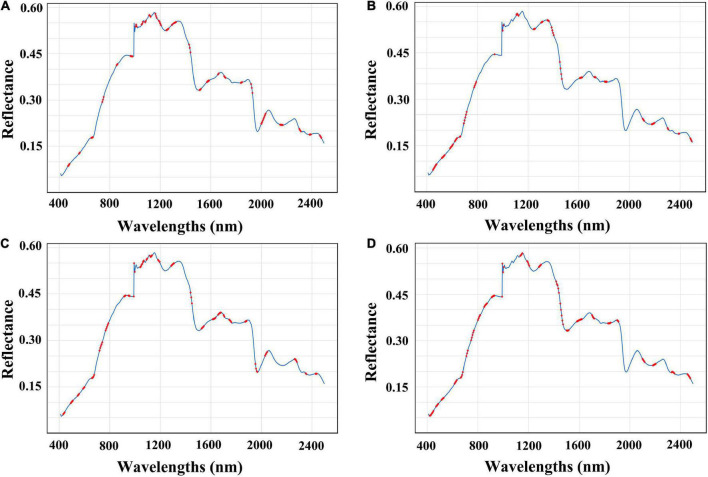
Selected wavelengths for best prediction in nutrient contents of RRB fruits. The selected wavelengths are shown in red dots. **(A)** Selected wavelengths in PPS prediction (CARS-ORI-PLSR); **(B)** selected wavelengths in RS prediction (CARS-MSC-PLSR); **(C)** selected wavelengths in TF prediction (VISSA-DER-PLSR); **(D)** selected wavelengths in TP prediction (CARS-MSC-PLSR).

In this study, the correlation between selected bands and predicted nutrients was analyzed. The effective absorption at 1,000–1,100 nm and 1,150–1,300 nm (Figure 3A) may be related to the second harmonic of O-H and the first harmonic of C-H combination in the polysaccharides, respectively (33). As for RS analysis (Figure 3B), the wavelengths at 1,385 nm corresponded to the C-H second overtone and combination as well as the wavelengths at 605 and 540 nm corresponded to the fourth and fifth overtone regions of –O-H from RS, respectively (34, 35). Meanwhile, the effective wavelengths of TF were almost consistent with former published studies, where 1,100 to 1,140,nm and 1,650 to 1,690 nm corresponded to the first overtone region and second overtone region of –CH_3_ from flavonoids, respectively (Figure 3C) (34). Also, the 1,430 to 1,450 nm corresponded to the second overtone region of –CH from polyphenols (36), and wavelengths intervals of 425–520 and 725–995 nm corresponded to the most abundant phenolic compounds of ferulic acid in RRB (Figure 3D) (37).

Additionally, the prediction of different nutrients may have a unique model tendency. One example is that potato starch content was predicted based on the selected HSI bands, and the same results showed that the selection methods of CARS, iterative variable subset optimization (IVSO), and VISSA have different prediction effects. The preferred CARS-SVM model achieved the best performance with the highest R^2^ and RPD values (27). In this study, the necessary work was conducted to compare and choose the most appropriate model for the prediction of different types of nutrients from RRB fruits.

In this study, the combination of HSI technology with the PLSDA model achieved a better result in the origin prediction of RRB fruits. Similar results were obtained in narrow-leaved oleaster (*Elaeagnus angustifolia*) fruit traceability by using HSI technology, and the PLSDA model achieved a higher discrimination accuracy (>99%) than that of the SVM group (13). Besides, a similar study on origin classification of Rhizoma Atractylodis Macrocephalae obtained the highest classification accuracy of 97.3% by using the PLSDA model (38). In the PGI origin discrimination of this study based on the HSI full wavelength, the OPLSDA model was suitable for pairwise comparison of origin traceability. Meanwhile, the good results in region discrimination by using the OPLSDA model from previous reports were listed, including Thai Hom Mali rice traceability (39) as well as the origin prediction of Huangjing from the PGI regions of Qingyang City, China (40).

During data collection using HSI technology, the random noises caused by equipment status and material characteristics such as uneven sizes and colors could be effectively eliminated by using pretreatment methods (30, 41). Also, the spectral derivatization noises can be well eliminated by DER, SEC, and SG methods (42). MSC is commonly used to remove the undesirable scatter effect caused by uneven sample sizes and morphologies (43). In fact, it is difficult to know which kind of noise plays a dominant role in this analysis. Therefore, there are no definite criteria, and trying-out is required in the specific application to select the best method for error elimination. In addition, the representativeness and uniformity of samples are very important for model prediction. For origin discrimination errors, one possible reason may be that inconsistent maturity, freshness, and surface cleanliness of RRB samples from JXDX regions led to the low sample representativeness and therefore the misjudgment in geographical origin classification.

## Conclusion

The nutrient content (PPS, RS, TF, and TP) indicated that the quality of RRB fruits had a significant difference (*P* < 0.05) related to the planting regions and could be predicted by using full HSI wavelengths assisted with chemometrics. These nutrient contents could be well predicted with the HSI effective wavelengths selected via CARS and VISSA methods, and the prediction effects were even better than those from full wavelength groups, indicating the potential application in fruit quality control. The combination of HSI technology with chemometrics was a promising method for RRB fruit traceability from multiple regions, and the samples from three PGI regions were efficiently classified through pairwise comparison with the OPLSDA model. All these findings show the promising application of HSI technology in the future as a rapid and nondestructive method to achieve quantification of nutrient contents and determination of origins for RRB fruits. Future studies will collect RRB samples from more diverse regions and consider more influential factors, including varieties, fruit maturity and freshness, as well as the regional cultivation practices, to find out their contribution to and influence on the origin traceability and quality prediction.

## Data availability statement

The raw data supporting the conclusions of this article will be made available by the authors, without undue reservation.

## Author contributions

YW: investigation, resources and writing – original draft. YeZ: writing – original draft and formal analysis. YY and JN: writing – review and editing. YyZ: methodology. TN: data curation and formal analysis. LH and JY: conceptualization, supervision, and funding acquisition. All authors have read and agreed to the published version of the manuscript.

## Abbreviations

AHXC: Xuancheng County Anhui Province
BPNN: Back-propagation neural network
CARS: Competitive adaptive reweighted sampling
CQSZ: Shizhu County Chongqing City
DER: First derivative
FJND: Ningde City Fujian Province
GDMZ: Meizhou City Guangdong Province
GXYL: Yulin City Guangxi Province
GZQDN: Qiandongnan Miao and Dong Autonomous Prefecture Guizhou Province
HSI: Hyperspectral imaging
JXDX: Dexing County Jiangxi Province
MSC: Multiplicative signal correction
OPLSDA: Orthogonal partial least squares discrimination analysis
ORI: Original spectrum
PGI: Protected geographical indication
PLSDA: Partial least squares discriminant analysis
PLSR: Partial least square regression
PPS: Pectin polysaccharides
R^2^: Square of the curve correlation coefficient
RMSET: Root mean square error of training set
RMSEP: Root mean square error of prediction set
RPD: Residual predictive deviation
RRB: Red raspberry
RS: Reducing sugars
SEC: Second derivative
SG: Savitzky-Golay filtering
SVM: Support vector machines
TF: Total flavonoids
TP: Total phenolics
VISSA: Variable iterative space shrinkage approach
YNKM: Kunming City Yunnan Province
ZJCA: Chun’an County Zhejiang Province
ZJLS: Lishui City Zhejiang Province.
