# Supplementary material for: Nutrient content prediction and geographical origin identification of red raspberry fruits by combining hyperspectral imaging with chemometrics
Source: Front Nutr. 2022 Oct 17;9:980095. doi: 10.3389/fnut.2022.980095 (PMC9642070; doi:10.3389/fnut.2022.980095)
Supplement: Supplementary file 1 [file Data_Sheet_1.docx]

Supplementary Material

# Supplementary Figures and Tables

## Supplementary Figures


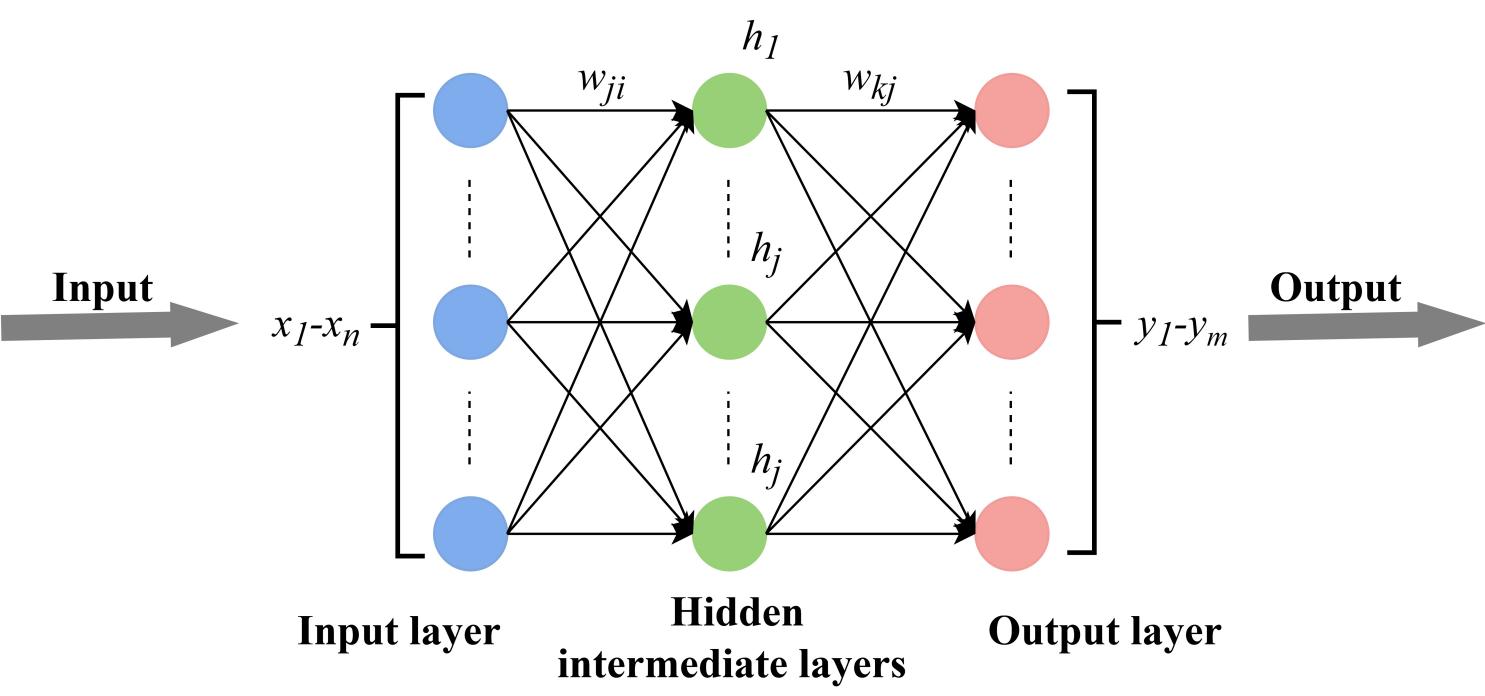


**Supplementary Figure 1.** The structure of the back-propagation neural network (BPNN) model. where ***x_1_-x_n_*** represents the input of the BPNN model; ***y_1_- y_m_*** represents the output of the BPNN model; ***h_j_*** represents the output of the hidden layers; ***w_ji_*** represents the weight values between the node number of the input layer (***i***) and the node number of the hidden layers (***j***); ***w_kj_*** represents the weight values between the node number of the hidden layers (***j***) and the node number of the output layer (***k***).


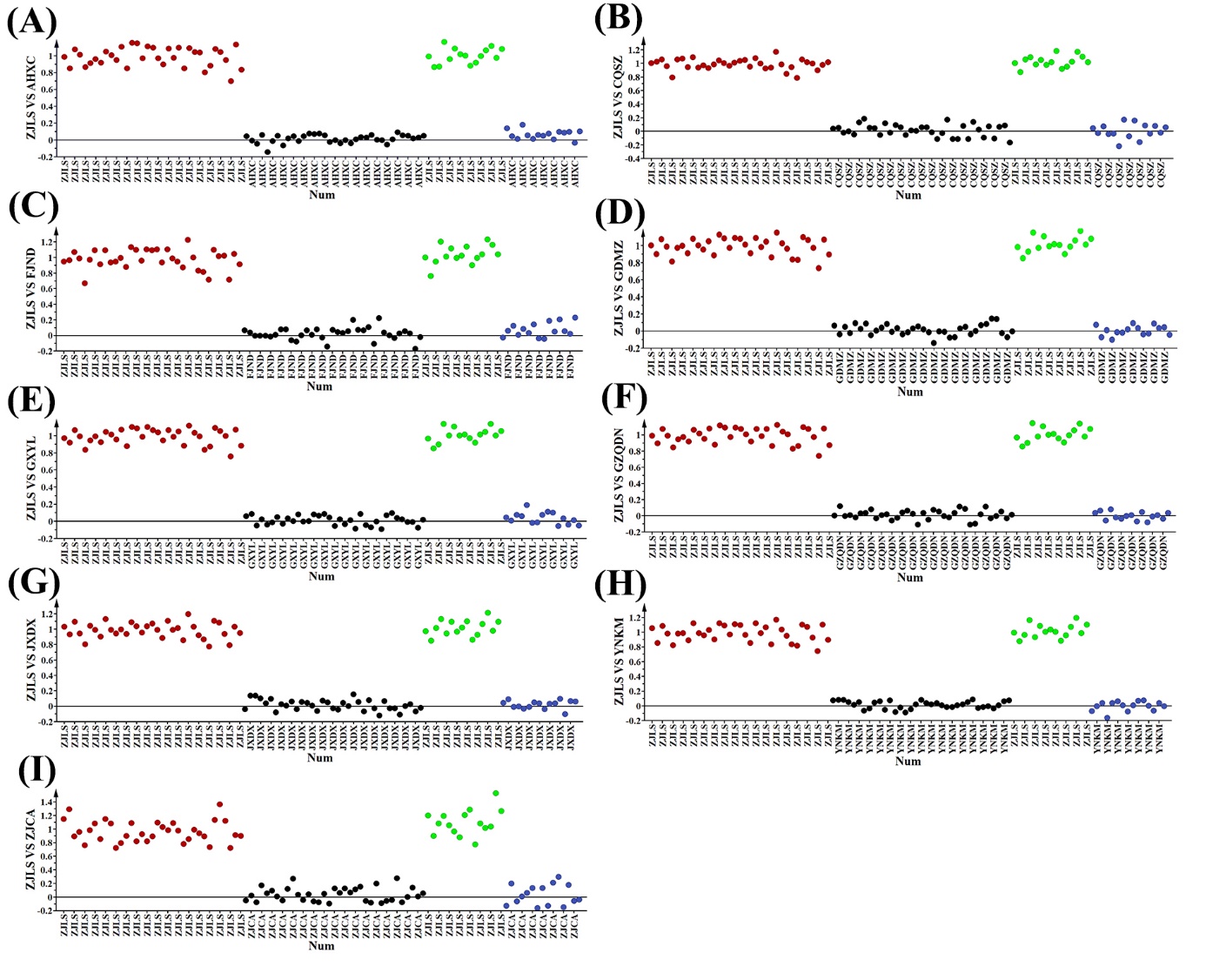


**Supplementary Figure 2.** Pairwise discrimination of RRB fruits from the PGI area of ZJLS based on full wavelengths. Ten production regions of RRB fruits including Xuancheng County, Anhui Province (AHXC); Shizhu County, Chongqing City (CQSZ); Ningde City, Fujian Province (FJND); Meizhou City, Guangdong Province (GDMZ); Yulin City, Guangxi Province (GXYL); Qiandongnan Miao and Dong Autonomous Prefecture, Guizhou Province (GZQDN); Dexing County, Jiangxi Province (JXDX); Kunming City, Yunnan Province (YNKM); Chun'an County, Zhejiang Province (ZJCA); Lishui City, Zhejiang Province (ZJLS). (A), ZJLS versus AHXC; (B), ZJLS versus CQSZ; (C), ZJLS versus FJND; (D), ZJLS versus GDMZ; (E), ZJLS versus GXYL; (F), ZJLS versus GZQDN; (G), ZJLS versus JXDX; (H), ZJLS versus YNKM; (I), ZJLS versus JZCA. The dots in red and black colors represent the training groups from protected regions and the other traditional production areas, respectively; the dots in green and blue colors represent the prediction groups from protected regions and the other traditional production areas, respectively. The same notations are used below.


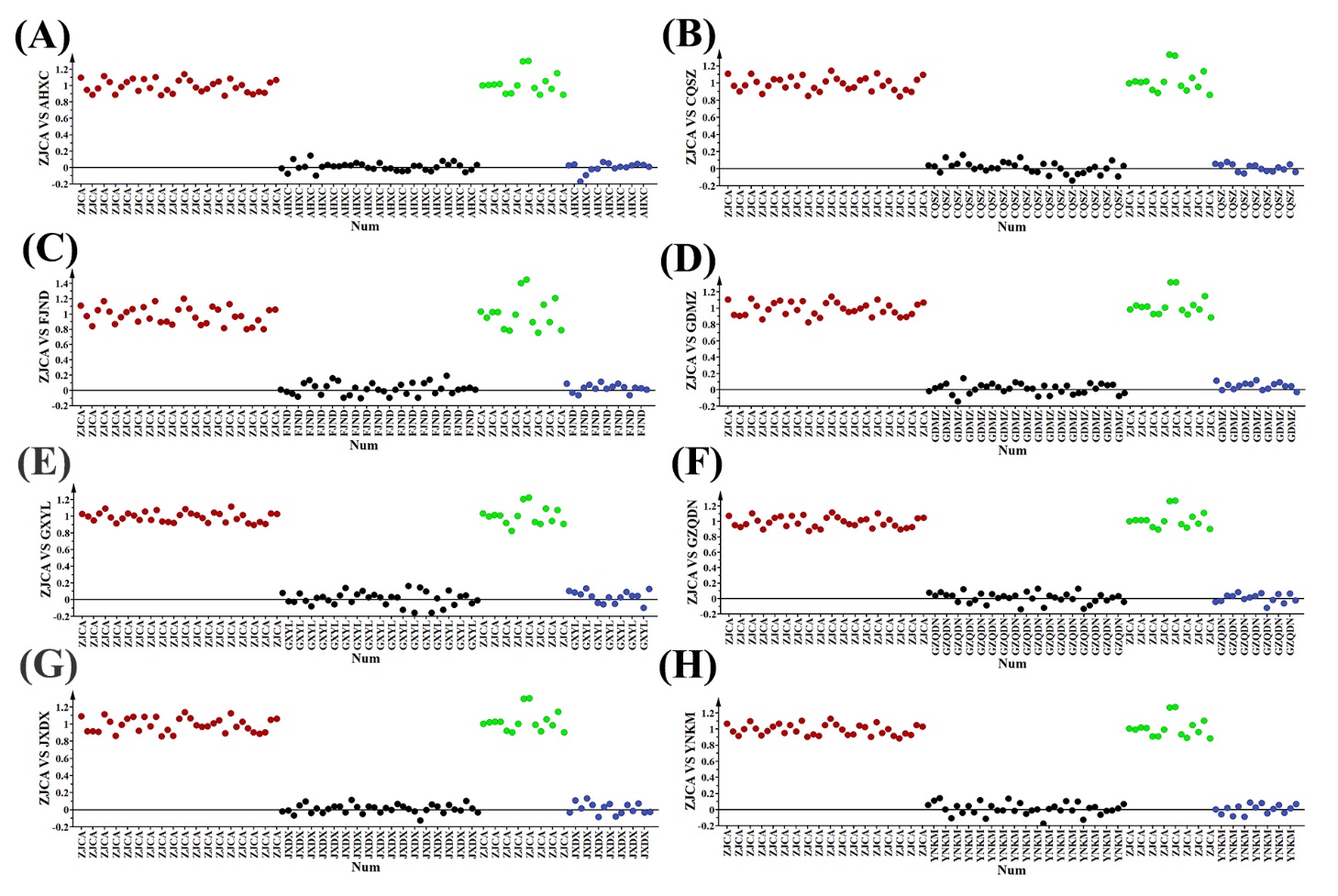


**Supplementary Figure 3.** Pairwise discrimination of RRB fruits from the PGI area of ZJCA based on full wavelengths. (A), ZJCA versus AHXC; (B), ZJCA versus CQSZ; (C), ZJCA versus FJND; (D), ZJCA versus GDMZ; (E), ZJCA versus GXYL; (F), ZJCA versus GZQDN; (G), ZJCA versus JXDX; (H), ZJCA versus YNKM.


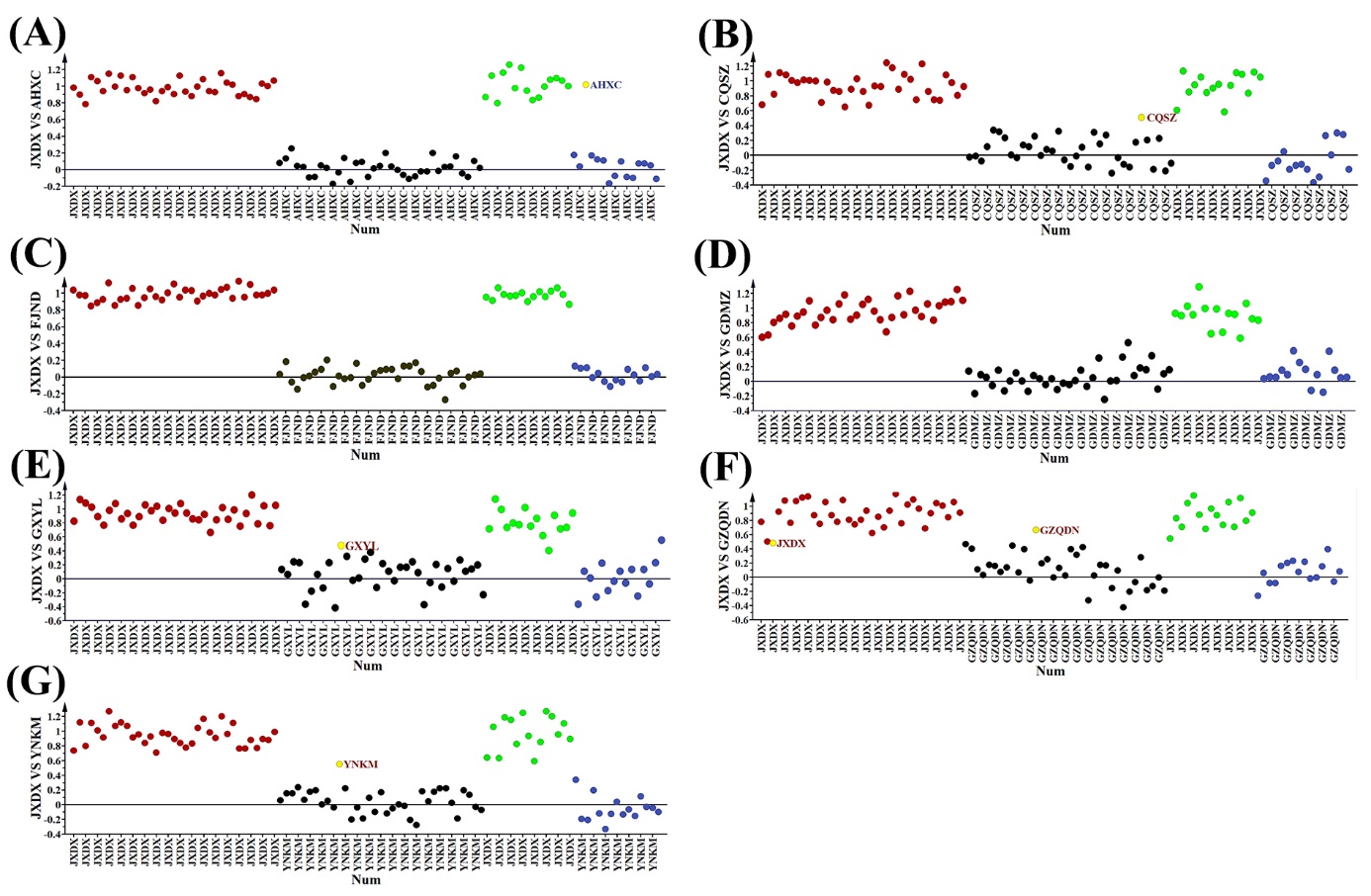


**Supplementary Figure 4.** Pairwise discrimination of RRB fruits from the PGI area of JXDX based on full wavelengths. (A), JXDX versus AHXC; (B), JXDX versus CQSZ; (C), JXDX versus FJND; (D), JXDX versus GDMZ; (E), JXDX versus GXYL; (F), JXDX versus GZQDN; (G), JXDX versus YNKM. The dots in yellow color represent the misjudgment in classification.

## 1.2 Supplementary Tables

**Supplementary Table 1.** Nutrient contents of RRB fruits cultivated in different regions.

| **Nutrients** | | **Regions** | | | | | | | | | |
| --- | --- | --- | --- | --- | --- | --- | --- | --- | --- | --- | --- |
|  |  | **AHXC** | **CQSZ** | **FJND** | **GDMZ** | **GXYL** | **GZQDN** | **JXDX** | **YNKM** | **ZJCA** | **ZJLS** |
| PPS (mg/g) | Max | 227 | 116 | 119 | 83 | 89 | 75 | 111 | 142 | 124 | 217 |
|  | Min | 178 | 101 | 105 | 73 | 68 | 63 | 93 | 126 | 103 | 164 |
|  | Mean | 203 ± 14.6^a^ | 107 ± 3.0^e^ | 112 ± 3.9^d^ | 77 ± 2.8^h^ | 81 ± 4.1^g^ | 70 ± 2.3^i^ | 101 ± 4.8^f^ | 135 ± 4.0^c^ | 115 ± 4.8^d^ | 179 ± 12.3^b^ |
| RS (mg/g) | Max | 310 | 230 | 290 | 240 | 270 | 225 | 230 | 280 | 260 | 339 |
|  | Min | 300 | 220 | 280 | 230 | 261 | 220 | 119 | 270 | 250 | 321 |
|  | Mean | 305 ± 3.4^b^ | 225 ± 3.4^h^ | 285 ± 3.2^c^ | 235 ± 3.0^g^ | 266 ± 2.9^e^ | 223 ± 1.6^i^ | 225 ± 3.3^h^ | 275 ± 3.3^d^ | 255 ± 3.3^f^ | 330 ± 5.4^a^ |
| TF (mg/g) | Max | 34.0 | 10.5 | 19.7 | 9.0 | 5.7 | 12.9 | 8.3 | 30.4 | 24.4 | 17.5 |
|  | Min | 20.9 | 5.4 | 12.3 | 3.4 | 3.0 | 7.5 | 3.5 | 18.0 | 16.9 | 10.2 |
|  | Mean | 28.1 ± 3.2^a^ | 8.4 ± 1.3^g^ | 15.9 ± 1.8^d^ | 5.6 ± 1.2^h^ | 3.3 ± 1.2^i^ | 10.8 ± 1.2^f^ | 6.2 ± 1.1^h^ | 24.6 ± 2.6^b^ | 21.3 ± 1.6^c^ | 14.0 ± 1.9^e^ |
| TP (mg/g) | Max | 11.6 | 13.8 | 13.1 | 13.0 | 11.8 | 12.7 | 14.2 | 12.1 | 12.8 | 11.1 |
|  | Min | 11.4 | 13.7 | 12.8 | 12.8 | 11.0 | 12.5 | 14.1 | 11.9 | 12.6 | 10.0 |
|  | Mean | 11.5 ± 0.06^h^ | 13.7 ± 0.03^b^ | 12.9 ± 0.08^c^ | 12.9 ± 0.05^d^ | 11.4 ± 0.15^i^ | 12.6 ± 0.06^f^ | 14.2 ± 0.03^a^ | 12.0 ± 0.03^g^ | 12.7 ± 0.05^e^ | 10.5 ± 0.28^j^ |

PPS, RS, TF, and TP indicate pectin polysaccharides, reducing sugars, total flavonoids, and total phenolics, respectively. Xuancheng County, Anhui Province (AHXC); Shizhu County, Chongqing City (CQSZ); Ningde City, Fujian Province (FJND); Meizhou City, Guangdong Province (GDMZ); Yulin City, Guangxi Province (GXYL); Qiandongnan Miao and Dong Autonomous Prefecture, Guizhou Province (GZQDN); Dexing County, Jiangxi Province (JXDX); Kunming City, Yunnan Province (YNKM); Chun'an County, Zhejiang Province (ZJCA); Lishui City, Zhejiang Province (ZJLS); Max, maximum value; Min, minimum value; Mean, average value with standard deviation; Lowercase letters a, b, c, etc. indicate a significant difference at the level of *P <* 0.05. The same notations are used below.

**Supplementary Table 2.** Prediction of pectin polysaccharides (PPS) content based on the full wavelengths.

| **Models** | **Pretreatments** | **Training set** | | **Prediction set** | | |
| --- | --- | --- | --- | --- | --- | --- |
|  |  | **R^2^** | **RMSET** | **R^2^** | **RMSEP** | **RPD** |
| BPNN | ORI | 0.833 | 40.25 | 0.760 | 22.19 | 1.98 |
|  | DER | 0.939 | 11.28 | 0.780 | 22.58 | 2.13 |
|  | MSC | 0.777 | 21.60 | 0.748 | 22.04 | 1.70 |
|  | SEC | 0.899 | 15.51 | 0.816 | 21.07 | 2.34 |
|  | SG | 0.892 | 14.80 | 0.806 | 19.81 | 2.23 |
| PLSR | ORI | 0.920 | 12.68 | 0.861 | 17.46 | 2.67 |
|  | DER | 0.906 | 13.76 | 0.851 | 17.44 | 2.58 |
|  | MSC | 0.931 | 11.82 | 0.881 | 15.48 | 2.89 |
|  | SEC | 0.901 | 14.11 | 0.855 | 17.15 | 2.58 |
|  | SG | 0.913 | 13.25 | 0.846 | 18.24 | 2.53 |
| SVM | ORI | 0.947 | 10.78 | 0.751 | 23.53 | 1.99 |
|  | DER | 0.999 | 1.08 | 0.850 | 17.46 | 2.55 |
|  | MSC | 0.971 | 7.83 | 0.777 | 21.27 | 2.06 |
|  | SEC | 0.999 | 1.08 | 0.816 | 19.37 | 2.29 |
|  | SG | 0.943 | 11.15 | 0.751 | 23.44 | 1.98 |

BPNN, back-propagation neural network; PLSR, partial least square regression; SVM, support vector machines; ORI, original spectrum; DER, first derivative; SEC, second derivative; SG, Savitzky-Golay filtering; MSC, multiplicative signal correction; R^2^, square of curve correlation coefficient; RMSET, root mean square error on the training set; RMSEP, root mean square error on the prediction set; RPD, residual predictive deviation. The same notations are used below.

**Supplementary Table 3.** Prediction of reducing sugars (RS) content based on the full wavelengths.

| **Models** | **Pretreatments** | **Training set** | | **Prediction set** | | |
| --- | --- | --- | --- | --- | --- | --- |
|  |  | **R^2^** | **RMSET** | **R^2^** | **RMSEP** | **RPD** |
| BPNN | ORI | 0.915 | 9.94 | 0.874 | 13.25 | 2.52 |
|  | DER | 0.957 | 7.06 | 0.887 | 12.66 | 2.93 |
|  | MSC | 0.875 | 11.93 | 0.842 | 15.20 | 2.10 |
|  | SEC | 0.954 | 7.23 | 0.827 | 15.96 | 2.38 |
|  | SG | 0.921 | 9.74 | 0.830 | 15.40 | 2.32 |
| PLSR | ORI | 0.872 | 12.01 | 0.882 | 13.12 | 2.67 |
|  | DER | 0.950 | 7.54 | 0.910 | 11.61 | 3.11 |
|  | MSC | 0.969 | 5.92 | 0.913 | 11.31 | 3.16 |
|  | SEC | 0.961 | 6.66 | 0.920 | 10.63 | 3.32 |
|  | SG | 0.963 | 6.47 | 0.925 | 10.27 | 3.48 |
| SVM | ORI | 0.778 | 14.69 | 0.514 | 23.41 | 1.28 |
|  | DER | 0.865 | 11.54 | 0.673 | 18.54 | 1.52 |
|  | MSC | 0.862 | 11.67 | 0.587 | 21.29 | 1.36 |
|  | SEC | 0.816 | 13.63 | 0.609 | 20.55 | 1.39 |
|  | SG | 0.760 | 15.34 | 0.506 | 23.46 | 1.24 |

**Supplementary Table 4.** Prediction of total flavonoids (TF) content based on the full wavelengths.

| **Models** | **Pretreatments** | **Training set** | | **Prediction set** | | |
| --- | --- | --- | --- | --- | --- | --- |
|  |  | **R^2^** | **RMSET** | **R^2^** | **RMSEP** | **RPD** |
| BPNN | ORI | 0.917 | 2.64 | 0.682 | 4.35 | 1.85 |
|  | DER | 0.933 | 2.08 | 0.715 | 4.56 | 1.85 |
|  | MSC | 0.943 | 2.65 | 0.820 | 3.49 | 1.97 |
|  | SEC | 0.927 | 2.30 | 0.714 | 4.38 | 1.77 |
|  | SG | 0.899 | 2.84 | 0.703 | 4.42 | 1.62 |
| PLSR | ORI | 0.907 | 2.58 | 0.829 | 3.31 | 2.29 |
|  | DER | 0.942 | 2.00 | 0.851 | 3.13 | 2.51 |
|  | MSC | 0.949 | 1.91 | 0.855 | 3.06 | 2.55 |
|  | SEC | 0.938 | 2.04 | 0.862 | 3.18 | 2.42 |
|  | SG | 0.938 | 1.92 | 0.839 | 3.23 | 2.37 |
| SVM | ORI | 0.927 | 2.33 | 0.690 | 4.46 | 1.60 |
|  | DER | 0.983 | 1.13 | 0.862 | 2.98 | 2.42 |
|  | MSC | 0.960 | 1.70 | 0.805 | 3.53 | 2.12 |
|  | SEC | 0.954 | 1.85 | 0.835 | 3.24 | 2.23 |
|  | SG | 0.925 | 2.36 | 0.671 | 4.60 | 1.54 |

**Supplementary Table 5.** Prediction of total phenolics (TP) content based on the full wavelengths.

| **Models** | **Pretreatments** | **Training set** | | **Prediction set** | | |
| --- | --- | --- | --- | --- | --- | --- |
|  |  | **R^2^** | **RMSET** | **R^2^** | **RMSEP** | **RPD** |
| BPNN | ORI | 0.947 | 0.238 | 0.852 | 0.468 | 2.56 |
|  | SG | 0.868 | 0.340 | 0.805 | 0.535 | 1.72 |
|  | DER | 0.949 | 0.227 | 0.874 | 0.412 | 2.65 |
|  | SEC | 0.951 | 0.223 | 0.887 | 0.388 | 2.88 |
|  | MSC | 0.919 | 0.299 | 0.894 | 0.376 | 2.85 |
| PLSR | ORI | 0.914 | 0.296 | 0.888 | 0.388 | 2.88 |
|  | DER | 0.930 | 0.267 | 0.915 | 0.346 | 3.19 |
|  | MSC | 0.974 | 0.163 | 0.953 | 0.252 | 4.40 |
|  | SEC | 0.947 | 0.232 | 0.917 | 0.332 | 3.33 |
|  | SG | 0.937 | 0.252 | 0.905 | 0.357 | 3.09 |
| SVM | ORI | 0.952 | 0.767 | 0.792 | 0.531 | 1.91 |
|  | DER | 0.999 | 0.021 | 0.921 | 0.335 | 3.11 |
|  | MSC | 0.993 | 0.833 | 0.818 | 0.496 | 2.11 |
|  | SEC | 0.997 | 0.060 | 0.885 | 0.391 | 2.74 |
|  | SG | 0.940 | 0.250 | 0.774 | 0.552 | 1.82 |
